# SaFiDe: Detection of saccade and fixation periods based on eye-movement attributes from video-oculography, scleral coil or electrooculography data

**DOI:** 10.1016/j.mex.2023.102041

**Published:** 2023-01-24

**Authors:** Samuel Madariaga, Cecilia Babul, José Ignacio Egaña, Iván Rubio-Venegas, Gamze Güney, Miguel Concha-Miranda, Pedro E Maldonado, Christ Devia

**Affiliations:** a Departamento de Neurociencia, Facultad de Medicina, Universidad de Chile, Chile; b Centro Nacional de Inteligencia Artificial, CENIA, Chile; c Biomedical Neuroscience Institute, BNI, Universidad de Chile, Chile; d Departamento de Anestesiología y Medicina Perioperatoria, Facultad de Medicina, Universidad de Chile, Chile; e Charité-Universitätsmedizin Berlin, Charitéplatz 1, 10117 Berlin, Germany; f Humboldt-Universität zu Berlin, Institut für Biologie, 10099 Berlin, Germany; g Bernstein Center for Computational Neuroscience Berlin, Humboldt-Universität zu Berlin, 10115 Berlin, Germany

**Keywords:** Eye movement measurements, Fixation, Saccades, Eye-tracking technology, Electrooculography, Saccade and fixation detection from eye-trace data (SaFiDe)

## Abstract

In this work we present SaFiDe, a deterministic method to detect eye movements (saccades and fixations) from eye-trace data. We developed this method for human and nonhuman primate data from video- and coil-recorded eye traces and further applied the algorithm to eye traces computed from electrooculograms. All the data analyzed were from free-exploration paradigms, where the main challenge was to detect periods of saccades and fixations that were uncued by the task. The method uses velocity and acceleration thresholds, calculated from the eye trace, to detect saccade and fixation periods. We show that our fully deterministic method detects saccades and fixations from eye traces during free visual exploration. The algorithm was implemented in MATLAB, and the code is publicly available on a GitHub repository.

• The algorithm presented is entirely deterministic, simplifying the comparison between subjects and tasks.
• Thus far, the algorithm presented can operate over video-based eye tracker data, human electrooculogram records, or monkey scleral eye coil data.

## Graphical abstract

**Figure:**
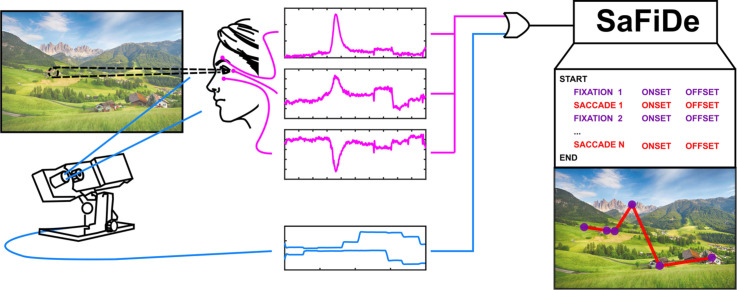

Specifications table

**Table:** 

Subject Area:	Neuroscience
More specific subject area:	Eye movement measurements
Method name:	Saccade and fixation detection from eye-trace data (SaFiDe)
Name and reference of original method:	Maldonado P, Babul C, Singer W, Rodriguez E, Berger D, Grün S. Synchronization of neuronal responses in primary visual cortex of monkeys viewing natural images. J Neurophysiol 100: 1523–1532, 2008.
Resource availability:	The method is implemented in Matlab code and publicly available in GitHub https://github.com/smadariagar/SaFiDe

## Introduction

### Background

Currently, the analysis of eye-tracking data has broad applications in science, medicine, and industry. In science, particularly in neuroscience and psychology, eye tracking is used to study human and nonhuman cognition [2] and essential cognitive processes in humans, such as reading and literacy acquisition [17]. Many other cognitive processing aspects can be studied through pupillometry, gaze position, and fixation/saccade and blinks; all measures are possible through eye-tracking techniques. In medicine, particularly in clinical practice, eye tracking has been used for skill training for procedures, such as laparoscopy, where training gaze behavior significantly improves medical trainees' performance [21]. Eye tracking has also been used to train surgery residents [6] and radiologists [20], among others [1], and is also broadly used as a research tool to support clinical diagnosis and training [5,10,18,19]. In industry, eye-tracking applications range from evaluating web advertising efficacy [15] to driving optimization [9].

Video-based eye-tracking devices compute behavioral and physiological features using eyes’ video recording. Software can process the images to identify pupillometry and, combined with corneal reflex, estimate gaze position; based on this, it detects periods of saccadic movements, fixations, and eyeblinks. Thus, the eye-tracking technique provides a rich amount of data useful for many applications. However, since it is based on image-video processing, it is highly dependent on environmental light conditions, making it difficult to record quality data outside a laboratory environment. Moreover, the behavioral and physiological measures drawn from eye-tracking studies can be expanded or complemented by the neural correlates of the cognitive process studied. The latter is mainly recorded through MEG (magnetoencephalography), EEG (electroencephalography), and intracortical recordings concurrently with eye-tracking recordings. Simultaneous recording of brain activity, eye movements, and pupil dynamics presents a unique opportunity to study the involvement of different brain structures in cognitive processes but also poses a technical challenge for the detection of saccades, fixations, and blinks.

It is necessary to develop more robust and systematic eye movement detection methods to study behavior in more ecological conditions. Specifically, (1) when environmental light conditions interfere with eye video recording and (2) when analyzing brain activity time-locked to saccade or fixation onset, an objective and reproducible eye movement detection method is needed.

### The proposed method

Here, we present a method to detect saccade and fixation periods based on eye-tracing features, and we apply it to human and nonhuman primate data. Eye traces can be computed either from eye tracking or from electrooculogram (EOG) data. The method we propose here was first used by our group to detect saccades and fixations from monkeys’ eye traces to study brain activity time-locked to eye movements [14]. In this work, we further develop this method to detect saccades and fixations from human and monkey recordings. Our approach has two main characteristics: (1) the features it uses are fully deterministic and computed from raw eye-trace data, making the results (e.g., saccade rate) comparable between subjects, thus increasing the reproducibility of the results; (2) it can be applied to human and nonhuman primate recordings. Additionally, it can be computed from video oculography or EOG records. In this work, we explain our detection method and apply it to human and nonhuman data during free-viewing tasks.

## Method details

### General idea of the method and the data used

**Fig. 1:**
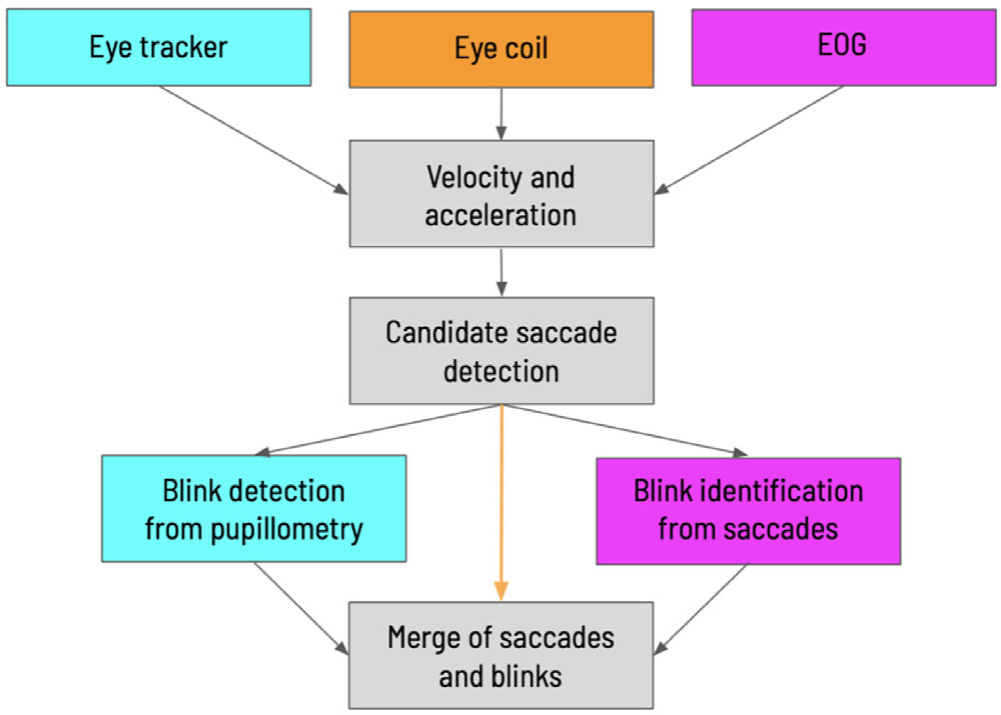
Schematic of the steps of the method developed to detect saccades and fixations from one eye. On gray, the core steps to identify saccades, on cyan processes specially implemented for eye-tracking records, on magenta processes adjusted for EOG data, and in orange, the steps performed to process eye coil recordings.

The method we developed uses the eye trace from one eye to detect saccades and fixation periods based on velocity and acceleration thresholds. It is publicly available on a GitHub repository. The input data can be from video eye tracking, scleral eye coils, or EOGs (Fig. 1). The core of the analysis is based on the velocity and acceleration of the eye movements, with different blink detection methods for each data input. In the next paragraphs, we describe the details of the analysis and provide the reasoning for the implemented solutions.

### Human recording

Human recordings were taken from two free-viewing tasks of natural images. The Ethics Committee for Human Research of Universidad de Chile approved both protocols.

In Experiment 1, Subject A freely explored natural scenes while EEG and video eye-tracking data were recorded (a female subject who had normal vision, was 28 years old, right-handed, and had right ocular dominance). The subjects explored several image types (plain gray, plain white, plain black, natural scenes on grayscale, and noise) freely. A total of 46 natural scenes from the IAPS database were used [12]. These stimuli images were interleaved with control images. A trial started with a blank screen presented for a period between 900 and 1500 ms, followed by a stimulus image explored for 3950 ms. In the current work, we further analyzed only eye traces when the subject was exploring natural scenes. Details of the methods are on [3].

In Experiment 2, Subject B freely explored natural images where, contingent on a saccadic eye movement, we introduced a Gabor patch of 1.5–2.5 visual degrees (the subject was a 33-year-old male with normal vision and right ocular and hand dominance). Stimulus presentation was developed in Python 2.7. We further analyzed the eye trace when the subject was exploring natural scenes (*n* = 80 trials).

The same general setup was used on both experiments. Subjects were sited 70 cm away from the screen. Images were presented on a screen of 1920 × 1080 pixels (32 px/cm), equivalent to 39.38 pixels per visual degree. Images were 1024 × 768 pixels in the center of the screen over a mid-gray background. Screen luminance was gamma-corrected. We recorded binocular eye movements with an eye tracker (Eyelink 1000, SR Research, Ontario, Canada) at a sampling rate of 500 Hz using the pupil area to collect pupillometry. To avoid head movement, the subject rested on a chin rest during recording.

### Nonhuman primates recording

We used eye traces from monkey D, a male capuchin monkey whose experimental data were already published by Maldonado et al. [14]. In each trial, the animal freely explored natural images for 3 s. Eye position was recorded by an eye coil implanted on the sclera of each eye. A calibration procedure was performed before each recording session. For the current analysis, we randomly chose the third experimental session; it had 20 trials of natural scenes composed of 15 different images.

### Eye trace collection

**Fig. 2:**
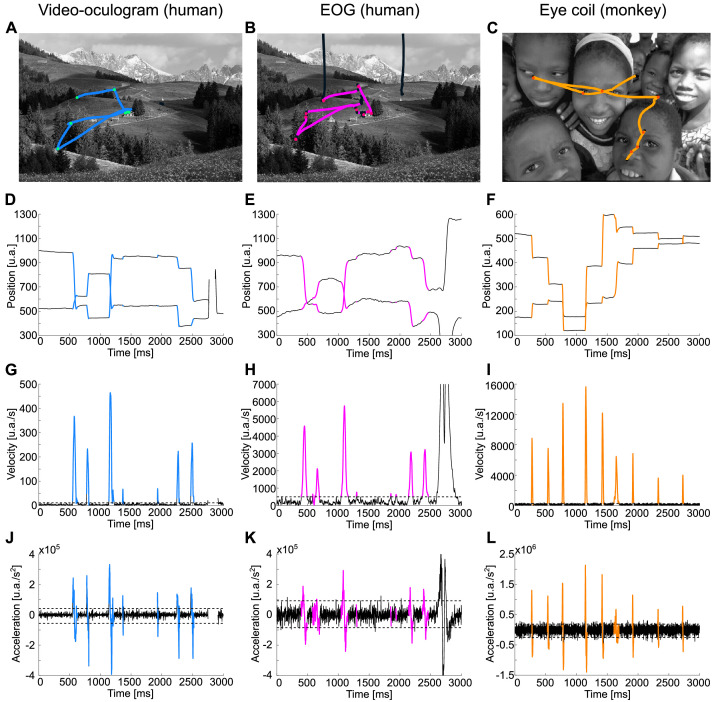
In the first row, the saccades are shown in color, and the colored dots represent the average position on the fixation. A. Scan path of one eye recorded by an eye tracker from one trial laid over an image from the human Subject A. B. Scan path of one eye obtained from EOG recorded simultaneously with A. C. Scan path of one eye recorded with scleral eye coil from a monkey during free visual exploration. D. Eye trace for the scan path on A, which depicts the gaze position in the horizontal (upper trace) and vertical axes (lower trace) over time. E. Eye trace from EOG. F. Eye trace from scleral eye coil recordings. G. and J. show velocity and acceleration for the eye trace on D, respectively. Panels H and K depict the velocity and acceleration computed from the EOG eye trace. On I. and L, we show the velocity and acceleration measured from eye coil records on F. Note that the raw eye trace from the eye tracker, from the eye coil, and from EOG are in arbitrary units.

SaFiDe is based on recordings of eye position while subjects explore a visual scene presented on a screen (Fig. 2A–C). Eye position can be obtained with video eye tracking. It can also be extracted from EOG recordings or recorded with a scleral eye coil in nonhuman primates. After calibration, the eye tracking device gives the position of the eyes over the screen in the horizontal (*xi*) and vertical (*yi*) axes on pixel units (Fig. 2D). In general, these data can be recorded from humans or nonhuman primates. Eye position can also be estimated from EOG recordings (Fig. 2E). In this case, the horizontal component of the movement is computed as the difference of the signals from the electrodes placed near the external canthus of each eye (*xi* = right-left). The vertical component of the movement corresponds to the difference between the signals recorded on the electrodes on the vertical plane below and above the eyeball (*yi* = below-above). Thus, without any further calibration, the eye trace computed from EOG signals is in the same units as EEG, Volts, needing a transformation based on a calibration procedure to represent displacement units. No calibration was performed on these data; to highlight this point, we present EOG data in arbitrary units (a.u.). Scleral eye coil records eye position as a magnetic deflection; similar to EOG recordings, and the calibration process transforms data on Volts to displacement units (Fig. 2F).

In summary, the eye position (*xi, yi*) that is used by SaFiDe as input data to identify eye movements can be collected with an eye tracker but could also be extracted from EOG or scleral eye coil. Once the representation of eye movement in the two-dimensional plane (*xi, yi*) is obtained, the method moves to the next step.

### Detection of candidate saccades and fixations

The core of the method is to compute saccades and fixations based on velocity (Fig. 2G–I) and acceleration (Fig. 2J–L) from the eye position on the horizontal (*xi*) and vertical (*yi*) axes. First, we compute the Euclidean distance (Eq. (1)) between successive data samples. If calibration and distance between eyes and the image are available, Euclidean distance can be transformed to visual degrees (using the equivalence between pixels on screen and visual degrees, pix2grad). In experiments that lack these data, such as when subjects walk with the recording system, the method can still detect saccades and fixations, but kinematic parameters will have relative values. Based on distance values, either on visual degrees or relative units, we compute velocity (Eq. (2)) and acceleration (Eq. (3)) for each sample.

(1) $$d_{i}=\sqrt{{\left(y_{i+1}-y_{i}\right)}^{2}+{\left(x_{i+1}-x_{i}\right)}^{2}}\;\left(pix\right),\;d_{i}=\frac{d_{i}}{pix2gr}\left({}^{\circ }\right)\;$$

(2) $$v_{i}=\frac{d_{i}}{t_{i+1}-t_{i}}\left({}^{\circ }/s\right)\;$$

(3) $$a_{i}=\frac{v_{i+1}-v_{i}}{t_{i+1}-t_{i}}\left({}^{\circ }/s^{2}\right)\;$$

**Fig. 3:**
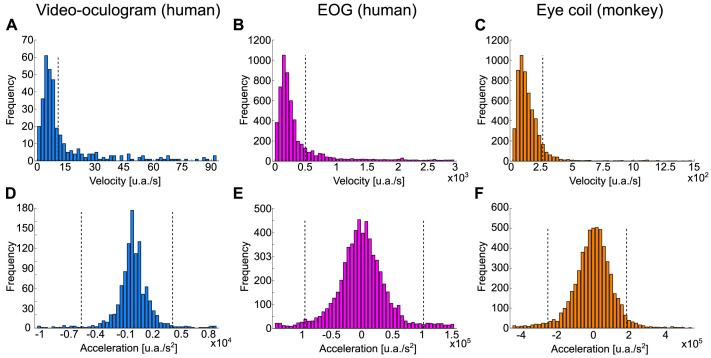
Threshold estimated from distributions of velocity and acceleration values. A. Histogram of the velocity samples computed with Eq. (2) from eye-tracker data. B. The same as A but from EOG recordings. C. Similar to A but computed on eye coil record from monkey. D. and E. Histogram of the acceleration computed with Eq. (3) on eye-tracking and EOG recordings, respectively. F. Same as E but for monkey data.

We defined a data sample as a saccade candidate if either velocity or the absolute value of acceleration were equal to or above the corresponding threshold (Fig. 3).

To set the thresholds, we consider the distribution of velocity (Fig. 3A–C) and acceleration samples (Fig. 3D–F); usually, they are a gamma distribution with a long tail. During free visual exploration of still scenes, most of the time, our eyes are fixated around a small area (roughly below 2°) and perform ballistic movements to reach other portions of the scene [22]. Our eyes perform roughly three or four fixations per second, lasting on average approximately 300 ms each fixation, but there is high variability across individuals and within individuals across different tasks [13]. Based on this, we can estimate that 80–90% of the time, our eyes move slowly around a small area. This characteristic means that on the velocity distribution, the long tail represents velocities while the eye is, probably, doing a saccade. As an example, in the observed velocity distribution in Fig. 3A, the velocity threshold was set at 14.3 a.u./s signaled by a dashed line. This threshold was also drawn as a dashed line in Fig. 2G, distinguishing periods of slow movements (black periods) from periods of high velocity (blue periods). Similar reasoning applies to the distribution of the acceleration; for all the data samples below -5600 a.u./s^2^ or above the 4160 a.u./s^2^ thresholds, the acceleration indicated that the sample was part of a saccade (comparing Fig. 3D dashed line with Fig. 2J dashed line).

**Fig. 4:**
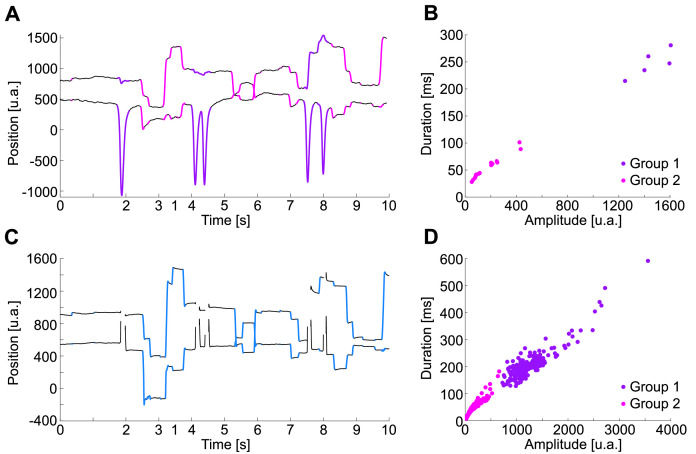
Blink identification from EOG data. A. Exemplary eye trace from human EOG recordings, Subject A, with saccades (magenta), fixations (black), and blinks (purple). Upper trace, eye gaze on the horizontal axis. Lower trace, eye gaze on the vertical axis. B. Main sequence of eye movements in A, amplitude versus duration. There is a visible separation between saccades (magenta) and the five blinks (purple). C. The same period that A recorded here with video eye tracking. D. Main sequence of half of the record (continues 30 min), EOG data, same color code that B.

The velocity and acceleration threshold can be determined by visual inspection or based on the literature [4]. To facilitate visual inspection, we implemented the code SaFiDe_thresholds.m, also available in the GitHub repository, which generates the velocity and acceleration distributions shown in Fig. 3. This code suggests the velocity and acceleration threshold using an empirical cumulative distribution function. The threshold was visually determined at 85% accumulation for velocity and 90% accumulation for positive and negative acceleration values. This threshold has been used in Fig. 2, Fig. 3, Fig. 4. For EOG data recorded from human subjects, the velocity threshold was 500 a.u./s (Fig. 3B), and the acceleration thresholds were -0.91 × 10^5^ and 1.05 × 10^5^ a.u./s^2^ (Fig. 3E). For eye coil data recorded from monkeys, the velocity threshold and acceleration thresholds were 249 a.u./s (Fig. 3C) and -2.53 × 10^5^ a.u./s^2^ and 1.86 × 10^5^ a.u./s^2^ (Fig. 3F), respectively. All these values were computed using the function SaFiDe_thresholds.

To summarize, based on the velocity and acceleration thresholds, we identified the samples that were candidates for eye movements. Then, a group of samples that exceed the thresholds and were successive in time are defined as periods of candidate saccade. Blinks also surpass the velocity and acceleration threshold; thus, the next step in the method is to detect blinks to make the candidate saccades compatible with blinks.

### Blink detection

During a blink, the eye gaze makes a fast vertical movement before the complete occlusion of the pupil by eyelid closure. Depending on the recording system, blink periods might be detected (Fig. 2D,E). In our case, blinks were recorded on video and EOG but were undetectable when recording scleral eye coils. Thus far, from the scleral eye coil, our method detects only saccades and fixations. To detect blinks, in the case of video-based eye tracking, we rely on a method based on pupillometry [8], while for EOG, we developed an amplitude-duration boundary based on the plot of the main sequence of the movement (Fig. 4).

Currently, video-based eye trackers record gaze position and pupillometry. To detect blinks here, we used a method based on video eye tracker noise estimation [8]. Hershman et al. proposed detecting blinks based on the variability of the first derivative of pupillometry. Their method has the advantage that it more precisely detects the onset and offset of blinks. On the other hand, from EOG recording, blinks are on a first stage identified as periods of saccade candidates. However, when the duration and amplitude of this candidate saccade were plotted on the main sequence diagram (Fig. 4B,D), the blinks were highlighted as data points with increased amplitude and duration, forming a separate cluster from actual saccades.

With periods of blinks detected on video or EOG, the next step in the method can be initiated in which the saccade and blink labels are corrected and merged to finally define fixation periods.

### Merge of saccades and blinks, and definition of fixations

The candidate saccades relabeled as blinks were discarded from the list of candidate saccades. Moreover, any candidate saccade with amplitude shorter than 0.1° or that lasted less than 4 ms was also excluded from the list. These thresholds are part of the parameters and can be changed by the user at SaFiDe code. Based on the final detected saccades and blinks, we labeled periods between saccades and between saccades and blinks or vice versa as fixations. Thus, fixations are defined as the periods when the eyes move slowly within a small area.

In summary, after blink detection, we actualized the list of saccades and discarded the shortest and defined periods of fixational eye movements. This merges all the movements on one disjoint list of timestamps, where each entry to the list depicts the time of movement onset and offset.

### Overshoot

**Fig. 5:**
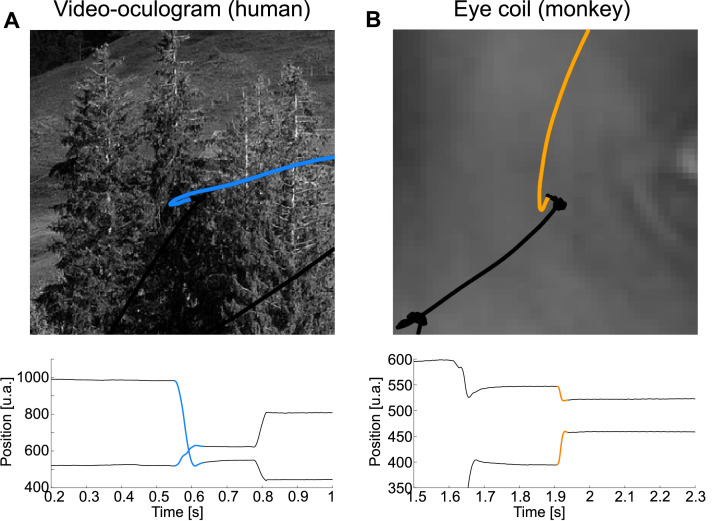
Examples of overshoot from human and monkey data. A. Example of overshoot from the eye tracker scan path; B. Example of overshoot on the monkey scan path.

We observed overshoots on human (Fig. 5A) and monkey (Fig. 5B) eye traces. Overshoots are corrective movements at the end of the ballistic movements of the saccade that correct the final position of the eyes. Technically, overshoots might be identified as another saccade because they represent a change in the movement direction, but they are actually part of the current saccade. Here, we consider the overshoot as part of the saccade because during this period, the eyes are still moving to reach their final position. SaFiDe detects both the first saccade and the correction saccade; then, considering that there is a single action, it unites them and puts a mark on the saccades with overshoot just in case that further analysis of those instances is needed. Specifically, we identify an overshoot when the time between two consecutive saccades was too short, below 16 ms, which is even below the shorter fixations reported at 50 ms [7]. For video eye tracking, the second saccade was small, with an amplitude below or equal to 1.5° These features indicate that the second saccade is a correction or redirection of the overshoot performed on the first part of the saccade (Fig. 5). Thus, the actual saccade's onset is when the first saccade starts, and the offset of the saccade is at the end of the overshoot. This definition for the offset of saccades after the end of the overshoot warrants a precise onset for fixations, just when the eye is performing slow movements over a central area.

### Brief method validation

We used two different methods to assess key aspects of SaFiDe performance. First, we performed a visual inspection to assess the identification of saccades done by the method. Second, we compare the saccades detected by SaFiDe on eye-trace data with those done over EOG records.

### Validation by visual inspection

**Fig. 6:**
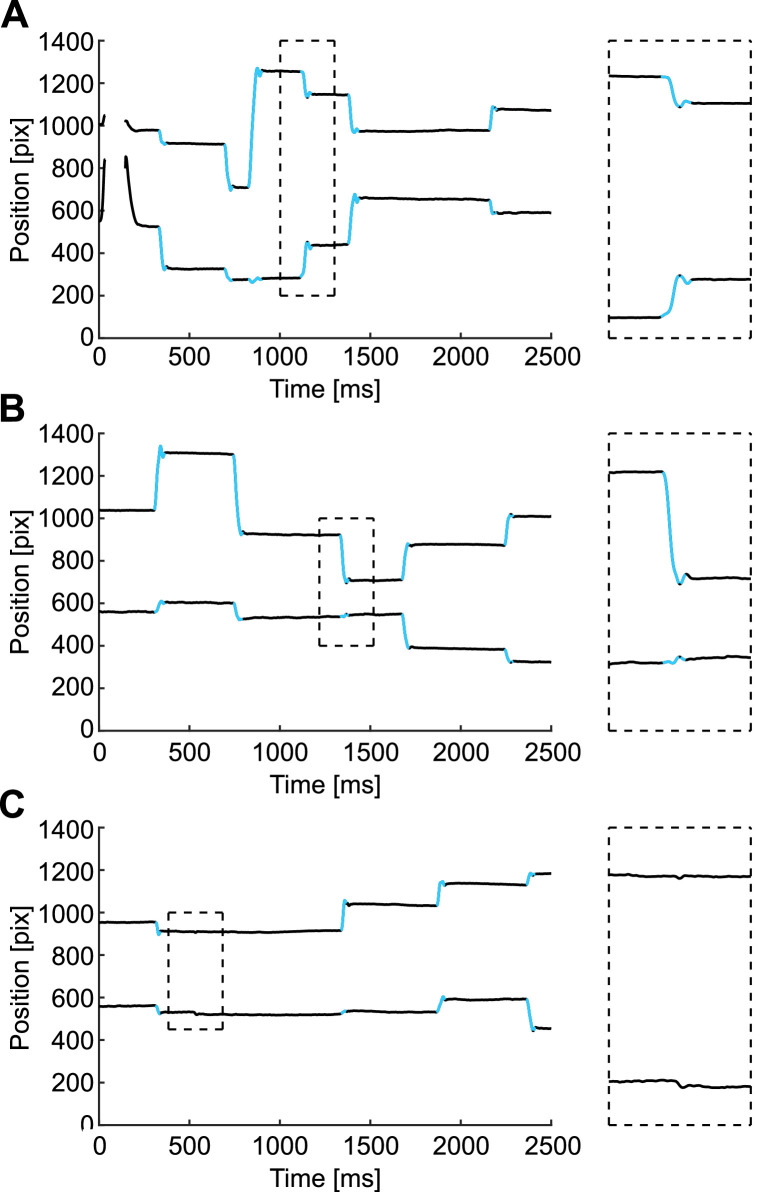
Three discrepancies in saccade identification between SaFiDe and human inspection. A and B are cases where an overshoot was detected by SaFiDe as a different movement from saccade. In Panel A, the third saccade has an overshoot that is detected as another saccade. The magnified image is on the right side of the panel. A similar case on the fifth saccade is shown in Panel B. On the other hand, as shown in Panel C, visual inspectors viewed a small saccade just after 500 ms, which was not detected by SaFiDe. Each panel shows on black the eye trace, the upper trace depicts the horizontal component of the eye movement and the lower trace represents the vertical component; on blue, the saccades detected by SaFiDe.

Two human judges who are familiar with eye movement theory and data (authors C.D. and S.M.) visually inspected the results from our algorithm and qualified its performance. We visually inspected 32 trials from Experiment 1 (experiment and subject description in Methods Details). With the current SaFiDe parameters (set by previous evidence: *velocity* > 30°/s; *acceleration* below −4000°/s^2^ or above 4000°/s^2^), the method detects 172 saccades; we visually verified 169 of them, equivalent to 98.3% of saccades. The extra saccades detected by SaFiDe corresponded to overshoots that were labeled as different saccades by the method (Fig. 6 Panels A and B). On the other hand, during the visual inspection, there was one case where both judges detected a small saccade that was not identified by SaFiDe with the current parameters (Fig. 6 Panel C).

**Fig. 7:**
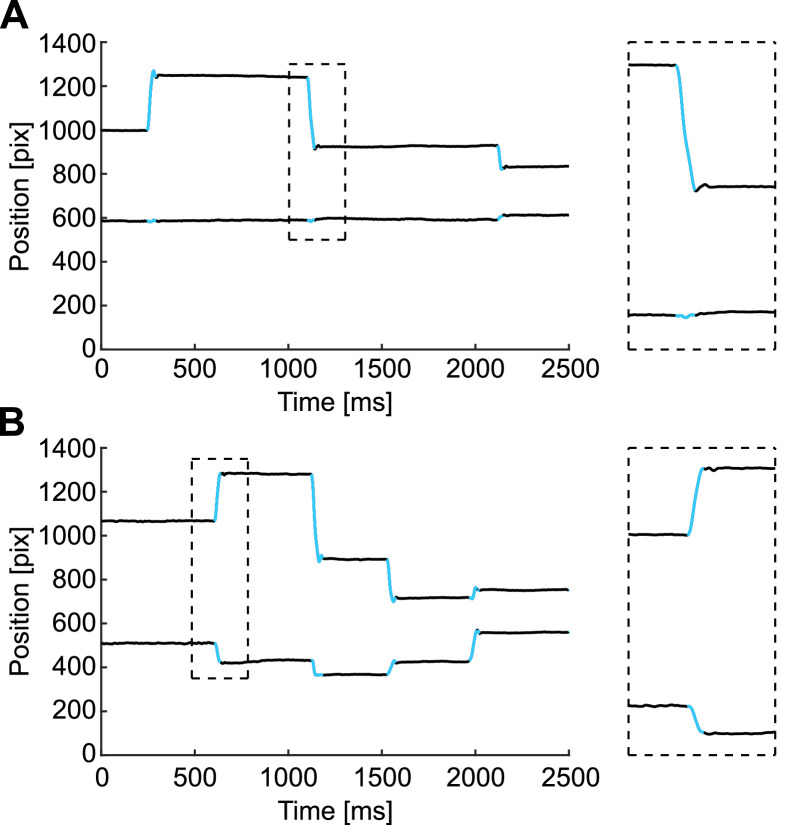
Observation of the onset and offset of saccades detected by SaFiDe. A and B show the saccades where the onset was one or two samples earlier than SaFiDe detected. Each panel shows on black the eye trace, the upper trace depicts the horizontal component of the eye movement and the lower trace represents the vertical component; on blue, the saccades detected by SaFiDe.

During the visual inspection, we observed that some saccades had their onset or offset one or two samples earlier or later than detected by SaFiDe. Overall, from the 172 saccades detected by SaFiDe, we observed in 6 of them that the detection was one or two samples later than our visual criteria, equivalent to 3.5% (Fig. 7 Panel A and B). The few discrepancies observed here should lead to an average error on the detection of ±3 ms.

Altogether, based on the visual inspection done, we are confident that the performance of SaFiDe is satisfactory. In our experience, the best parameters for SaFiDe are those suggested by the function SaFiDe_threshold, but on these validations, we used parameters based on previous evidence. In any case, a visual inspection should always be used to confirm the results.

### Validation by comparison

To assess the congruence between the saccades detected by SaFiDe from EOG and from video eye-tracking data, we compared the number of eye movements detected from each data source. To perform the detection, we automatically set the threshold with the function SaFiDe_threshold. Table 1 shows the results for the analysis of video eye-tracking data with SaFiDe and shows the average number of saccades detected per image over 80 images analyzed (parameters obtaining SaFiDe_thresholds: *velocity* > 19.21°/s, *acceleration* below −7000°/s^2^ or above 5350°/s^2^). The second row shows the results for the analysis of EOG recordings (SaFiDe_thresholds: *velocity* > 2730 µV/s, *acceleration* below −248,000 µV/s^2^ or above 258,000 µV/s^2^). The mean number of saccades per image depends on the method used (first row). This discrepancy relies on the low number of short saccades (<1°) detected from EOG data. When we consider only saccades from video eye-tracking data longer than 1°, the mean number of saccades per image decreases (second row); thus, reducing the difference in detection between the EOG and video eye-tracking data. Improving the signal-to-noise ratio on EOG recordings helps to increase the number of detected short saccades.

**Table 1:** Comparison of saccades detected by EOG and video eye tracking.

	EOG mean (std)	Video eye-tracking mean (std)	Difference mean (std)
Saccades per image	8.7 (2.8)	12.2 (3.0)	3.5 (2.5)
Saccades (> 1°) per image	Not available (needs calibration data)	10.7 (3.0)	2.1 (2.4)

## Conclusion

In this work, we present SaFiDe, an automatic fully deterministic method to detect saccades and fixations from eye trace data collected during free viewing. Our work was motivated by the lack of a deterministic method that could be applied to human and nonhuman primate data. With the SaFiDe method, the parameters of the detector are fully available to the experimenter and are the same between subjects. It uses velocity and acceleration thresholds to determine periods of saccades and fixations, and it can be applied over EOG, video eye-tracking, or scleral eye coil records. Moreover, for video and EOG data, we implemented a module to detect blink periods based on pupillometry [8] or data statistics, respectively. Validation of the method shows good performance in detecting saccades, even in the presence of overshoots.

From the beginning of eye-tracking techniques, the detection of saccades and fixations has been an important stage of analysis, mainly because information about where, when and how long a subject observes an area of a visual scene provides insight into the subject's mental dynamics [11]. Early on, to detect saccade onset and offset the velocity and/or acceleration threshold was used [4]. However, with the improvement of computer capacity, other methods have also been developed to detect the temporal limits of saccades [16]. In this work, we detected saccades based on velocity and acceleration thresholds. The main advantage of our method is its deterministic nature, its reproducibility across subjects, and the clear offset of the saccade. These results imply that analysis of brain activity time-locked to either saccade or fixation onset is precise and reproducible across subjects and setups.

In the future, we would like to test the performance of this method on other more ecological conditions, as with subjects walking on the street, and in other animal models. It would also be useful to test SaFiDe robustness and limits using synthetic data.

## Declaration of Competing Interest

The authors declare that they have no known competing financial interests or personal relationships that could have appeared to influence the work reported in this paper.

## Data Availability

Data will be made available on request.
